# Lipoma arborescens in a 16‐year‐old male: A case report

**DOI:** 10.1002/ccr3.5230

**Published:** 2021-12-18

**Authors:** Paa Kwesi Baidoo, Frank Nketiah‐Boakye, Emile Kouakou Tano, Majeedallahi Al‐Hassan, Gaddiel Obo Mensa Yorke, Ronald Awoonor‐Williams, Ernest Boakye

**Affiliations:** ^1^ Directorate of Orthopedics and Trauma Komfo Anokye Teaching Hospital Kumasi Ghana; ^2^ Department of Surgery Komfo Anokye Teaching Hospital Kumasi Ghana; ^3^ Department of Pathology Komfo Anokye Teaching Hospital Kumasi Ghana

**Keywords:** benign, intra‐articular, lipoma arborescens, tumor

## Abstract

Lipoma arborescens is a benign intra‐articular tumor characterized by joint effusions, pain, and reduced range of motion. It is rare in adults and children. We present a case of lipoma arborescens in a 16‐year‐old male. The work up involved plain radiographs, MRI, incisional biopsy, and laboratory analysis.

## Graphical abstract

Lipoma arborescens, though rare, should be considered as one of the many differential diagnoses in children presenting with knee swelling and chronic pain.

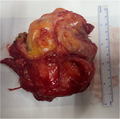

## 1 INTRODUCTION

Lipoma arborescens (L.A) is an uncommon benign intra‐articular lesion consisting of villous synovial hypertrophy and proliferation of mature fat cells.^1^ The term arborescens meaning “tree‐like” in Latin, describes the unique villous and frond‐like appearance of this condition.^2^ The knee is the most frequently involved joint and mostly unilateral.^3^ The etiology is unknown and usually presents in adults and is, however, rare in children.^4^, ^5^, ^6^, ^7^, ^8^ Even though the condition is uncommon, it should be considered as a differential diagnosis when patients present with chronic atraumatic knee pain and effusion.^9^ We present a case of lipoma arborescens in the left knee of a 16‐year‐old boy and discuss the clinical and laboratory workup and subsequent management.

## 2 CASE REPORT

A 16‐year‐old Ghanaian male presents to the outpatient clinic with a 5‐year history of recurrent atraumatic left knee pain and swelling which has worsened over the past one year. The pain was described as achy, about 8/10 on the visual analog scale, and localized mainly in the anteromedial and anterolateral aspect of the left knee. His symptoms were aggravated by activity and was also associated with a limp and knee stiffness. He did not have any pain in the left hip joint, groin, or any other joint, and there was no history of injury to the left lower limb previously. There has also not been any recent weight loss, anorexia, fever, or night pain.

On physical examination, he was afebrile, not pale and well nourished, and had a body mass index of 25 kg/m^2^. The left knee was swollen, tender, and was associated with a 15 cm by 15 cm doughy mass mostly localized to the suprapatellar region that was not fixed to the overlying skin or underlying structures. The distal pulses and sensation were all intact and comparable with the contralateral limb. The range of motion (ROM) in the left knee was 0°–40° compared to 0°–140° in the right knee. There were not ligamentous laxity. The following differential diagnosis were entertained based on the clinical findings; lipoma arborescens, myxoid lipoma, pigmented villonodular synovitis, synovial chondromatosis, liposarcoma, and synovial hemangioma.

**FIGURE 1:**
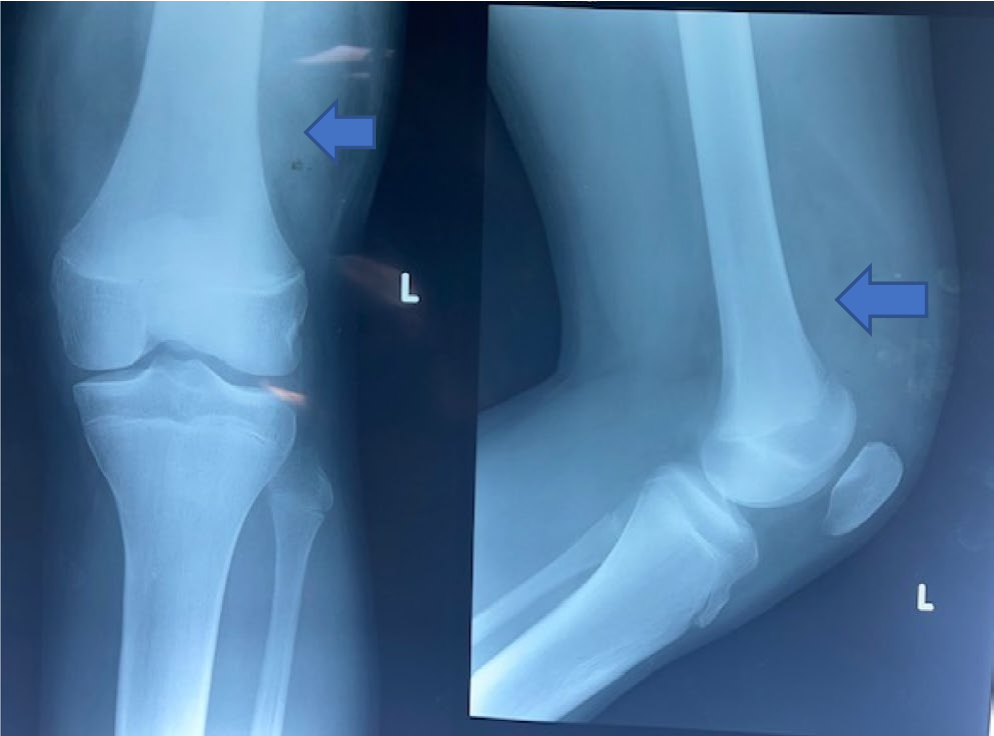
Anteroposterior (AP) and lateral views of the left knee showing normal bony appearance and extensive soft tissue swelling (blue arrow)

X‐rays of the left knee and pelvis showing both hip joints were obtained. The hips were essentially normal. X‐ray of the knee showed normal joint space, bony morphology and alignment, and no fractures or dislocations. There was, however, an extensive soft tissue mass in the suprapatellar region (Figure 1).

Laboratory test showed normal complete blood count (CBC), an elevated erythrocyte sedimentation rate (ESR) of 45 mm/h (normal range – 0–15 mm/h), normal values for C‐reactive protein, rheumatoid factor, and anti‐cyclic citrullinated peptides (Anti‐CCP).

**FIGURE 2:**
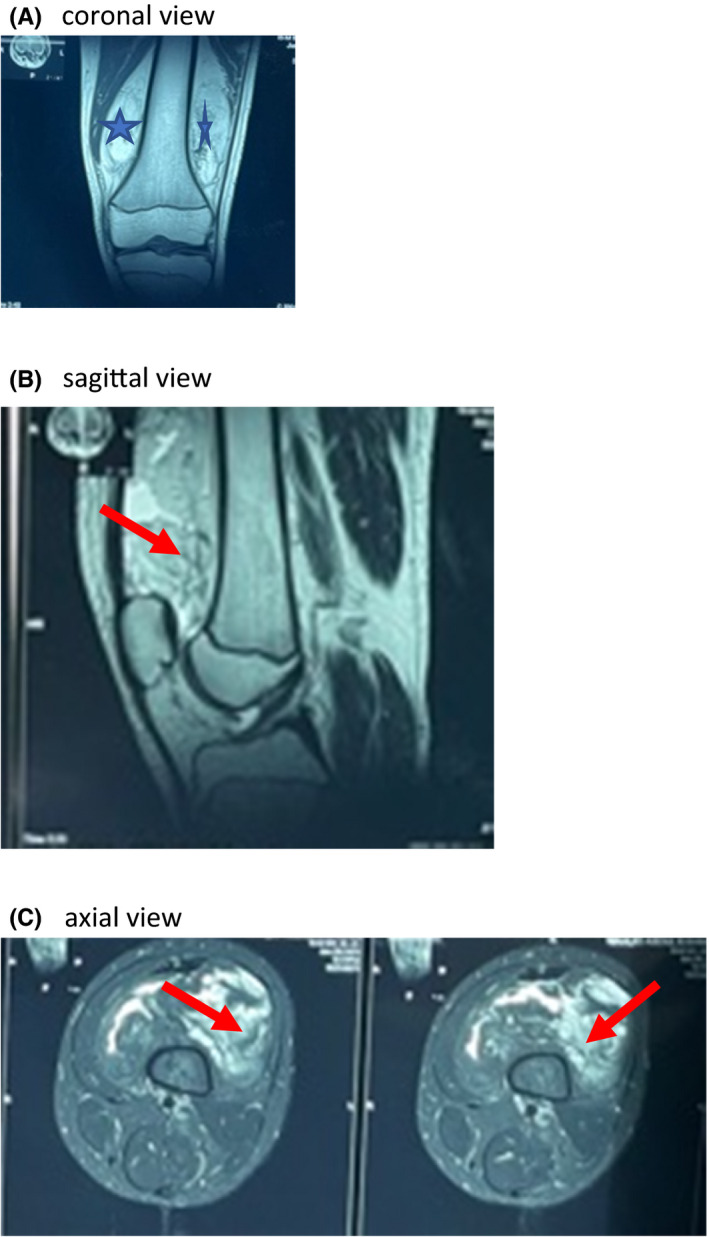
(A) T1‐weighted coronal images showing large fat‐signal mass in the lateral and medial aspect of the distal femur (star). (B) T1‐weighted sagittal image compatible with fat in the posterior aspect of the distal thigh and knee. (C) Axial views of the knee showing extensive feathery‐like lipoma arborescens (red arrows) in the suprapatellar region

Magnetic resonance imaging (MRI) with and without contrast showed a 5.6 cm × 8.8 cm × 10.1 cm heterogeneously enhancing lesion in the distal third of the left thigh, predominantly anterior and deep to the rectus femoris muscle. The lesion appeared is iso‐intense to the subcutaneous fat on T_2_W, hyperintense on T_1_Pd and suppresses on STIR. It also showed some areas of T_1_ hypointensities and T_2_/STIR hyperintensities which enhances post contrast. The lesion was seen to splay the adjacent muscles with no obvious infiltration of the muscles (Figure 2). The MRI was suggestive of liposarcoma and recommended a biopsy for confirmation. All other structures in the knee were essentially normal.

**FIGURE 3:**
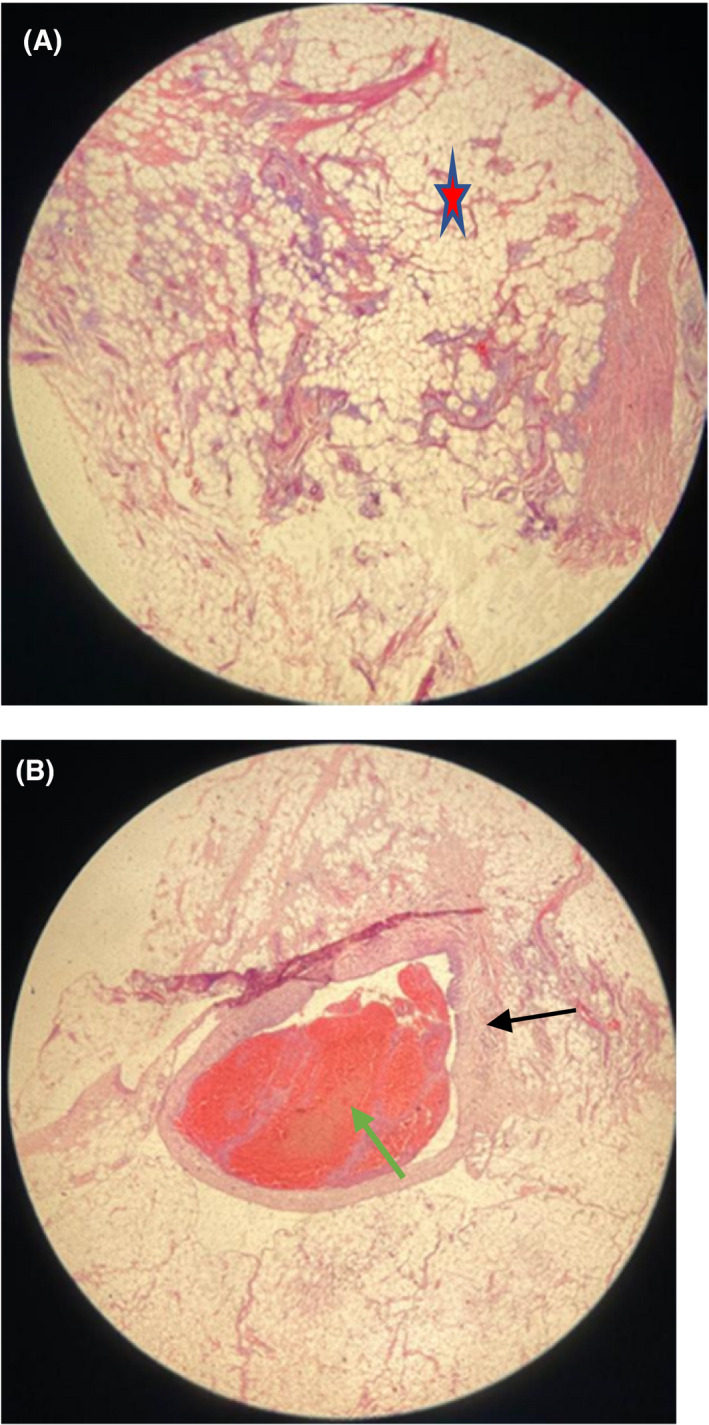
Histopathology showing synovial proliferation and matured lipocytes (red star) arranged in lobules (A), and an area containing thick walled blood vessel (black arrow) with thrombus(green arrow) (B)

The patient was worked up for an incisional biopsy following informed consent from the legal guardian. The specimen was sent for histopathology, the report of which indicated a lesion with focal papillary‐like fronts (hypertrophic villous projections of fat that is lined by synovial cells), scattered chronic inflammatory cells, and increased capillary blood vessels within the specimen and focal areas of fibrosis. The dominant cell types were matured fat cells arranged in lobules. This was consistent with lipoma arborescens (Figure 3).

**FIGURE 4:**
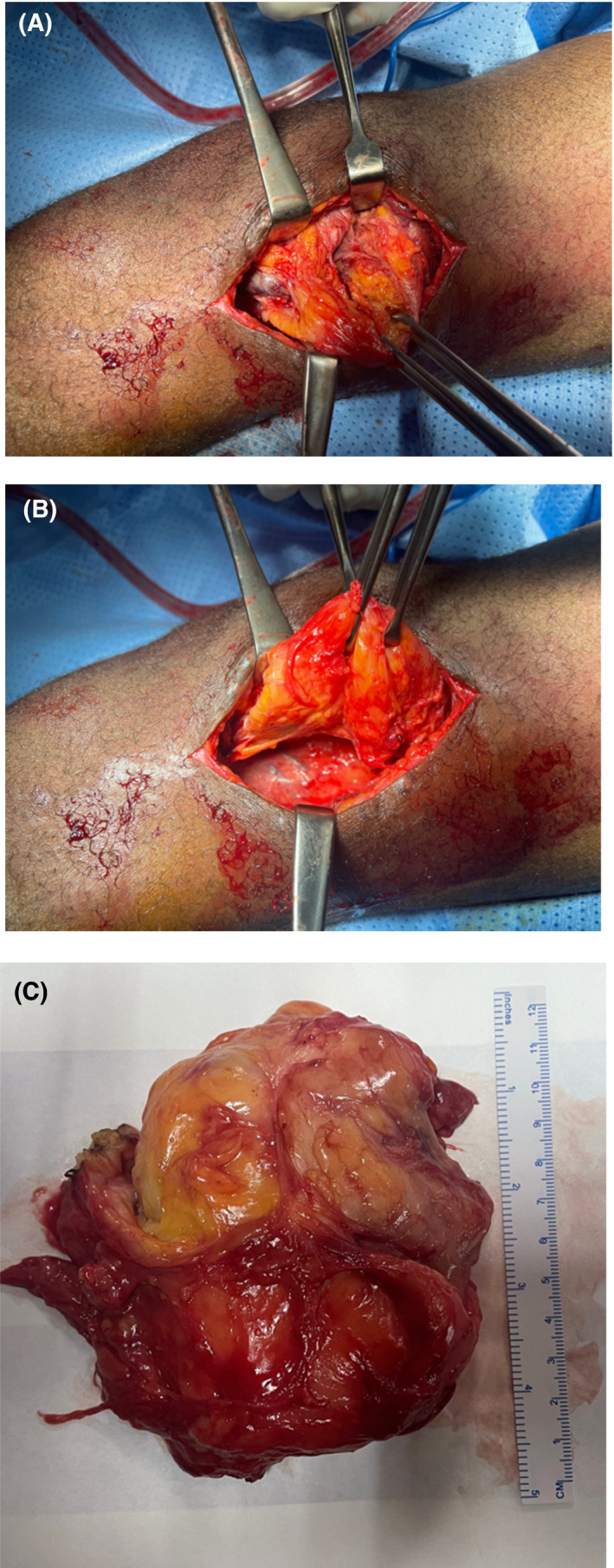
Intra‐operative pictures showing the lipoma arborescens in situ (A and B) and the post excision lesion (C)

A total open synovectomy and excision of the hypertrophic multilobulated yellowish white mass in the suprapatellar and medial recess was done 6 weeks later. The mass weighed 200 g and measured about 10 cm by 10 cm (Figure 4A–C). The specimen sent for histopathology again was consistent with lipoma arborescens. The patient was allowed to mobilize on the left lower limb a day after the surgery and also commenced physiotherapy to improve the range of motion of the left knee.

At 3‐month follow up, the patient was able to fully flex and extend the left knee with the range of motion been comparable with the right knee. There was also resolution of the swelling and the knee pain.

## 3 DISCUSSION

Lipoma arborescens is a very rare intra‐articular lesion which is characterized mainly by replacement of subsynovial tissue by matured fat cells and fibrous tissues given the unique villous transformation of the synovium into a tree‐like pattern.^1^ The condition was first described by Albert Hoffa, a German surgeon in 1904, and in 1957, Arzimanoglu described the lesion in much detail.^10^, ^11^ It commonly affects the knee^3^ but there has been reported cases in the shoulder,^12^ elbow,^13^ wrist,^14^ hip,^15^ and in synovial sheath and bursae which are extra‐articular.^16^

There has been fewer than 100 cases reported in the literature,^17^ of which fewer than 15 were children, making it extremely rare in this age group.^4^, ^6^, ^7^, ^8^, ^9^ The peak incidence is estimated to be between the third and fifth decades with a male predominance.^18^ Sanamandra et al,^2^ however, did not observe any sex preponderance. Though the etiology remains unknown, there has been reported association with trauma, degenerative or inflammatory joint diseases.^16^

Lipoma arborescens can be classified as either primary or secondary depending on the underlying condition or age of presentation.^19^, ^20^ The primary form of the lesion is usually idiopathic and are found in younger patients aged between 20 and 30 years.^16^ The secondary form which is more common and mostly seen in older patients is characterized by synovial lipomatosis as a result of chronic irritation of the synovium secondary to degenerative diseases, trauma, infections such as septic arthritis and meniscal injury.^2^

Patients with LA can present as in our patient, with a slow progressive swelling, recurrent joint effusion, restricted range of motion in the affected joint, and locking and pain without any history of trauma. On physical examination, there may be joint effusion and a palpable doughy mass mostly in the suprapatellar region.

Lipoma arborescens in the pediatric population should be differentiated from other atraumatic chronic knee swellings such as juvenile rheumatoid arthritis, Lyme disease, acute rheumatic fever, hemophilia, mycobacteria tuberculosis, pigmented villonodular synovitis, synovial osteochondromatosis, and synovial hemangiomas. CBC, ESR, C‐reactive protein, rheumatoid factor, anti‐CCP, and screening for Lyme disease in endemic areas should be done as part of the laboratory investigations to rule out the other differential diagnosis though these are nonspecific.

Plain x‐ray may show non‐specific soft tissue swellings, bone erosions, and osteoarthritic changes in older patients.^12^, ^16^ A frond‐like hyperechoic mass that waves during manipulation of the knee is observed on ultrasound scan.^21^ Computer tomography (C.T.) scan may show a villous synovial mass, the density of which is similar to fat with no enhancement when contrast is administered.^17^ MRI, which is the imaging modality of choice, is used to differentiate LA with its peculiar appearance from other intra‐articular masses.^16^ It appears as a large frond‐like mass arising from the synovium with similar signal intensity as subcutaneous fat on all pulse sequence.^16^ After pre‐saturation of the fat or signal suppression on short T1 inversion recovery (STIR) sequence, the subsynovial portion shows a high signal intensity on both T1 and T2 weighted images.^12^, ^19^ With the exception of the synovium layer and joint fluid which enhances due to the presence of inflammatory cells, the mass does not show enhancement with contrast.^22^ In addition to the features above, there may be different degrees of joint effusion. These characteristic features were all present in our patient.

Macroscopically, the lesion appears as a yellowish white mass with frond‐like pattern. Histologically, there may be papillary proliferation of synovial villi and substitution of subsynovial tissue by mature fat cells. The overlying synovium contains mononuclear infiltrate and synovial cells are enlarged and reactive with abundant eosinophilic cytoplasm.^3^, ^12^ Lipoma arborescens is mostly treated either by arthroscopic or open excisional biopsy depending on the extent of the lesion and the surgeon's personal experience. Recurrence after surgical excision is uncommon.^16^

## 4 CONCLUSION

Lipoma arborescens is a rare intra‐articular lesion that is characterized by villonodular proliferation of the synovium. Its unique appearance on MRI helps in differentiating it from other similar conditions. It can be treated safely and effectively with either open or arthroscopic synovectomy with low risk of recurrence.

## CONFLICT OF INTEREST

None declared.

## AUTHOR CONTRIBUTIONS

PKB, MA, GOMY, RAW, FNB, and EKT were directly involved in the surgery, follow up, and preparation of the manuscript. EB was responsible for reporting on the histopathology and also preparation of the manuscript.

## ETHICAL APPROVAL

Written informed consent was obtained from the legal guardian of the patient for publication of this case report and the associated images.

## Data Availability

Data sharing is not applicable to this article as no new data were created in this study.
